# Heat shock protein 90 is required for sexual and asexual development, virulence, and heat shock response in *Fusarium graminearum*

**DOI:** 10.1038/srep28154

**Published:** 2016-06-16

**Authors:** Duc-Cuong Bui, Yoonji Lee, Jae Yun Lim, Minmin Fu, Jin-Cheol Kim, Gyung Ja Choi, Hokyoung Son, Yin-Won Lee

**Affiliations:** 1 Department of Agricultural Biotechnology, Seoul National University, Seoul 08826, Republic of Korea; 2 Division of Applied Bioscience and Biotechnology, Institute of Environmentally Friendly Agriculture, College of Agriculture and Life Sciences, Chonnam National University, Gwangju 61186, Republic of Korea; 3 Eco-friendly New Materials Research Group, Research Centre for Biobased Chemistry, Division of Convergence Chemistry, Korea Research Institute of Chemical Technology, Daejeon 34114, Republic of Korea; 4 Centre for Food and Bioconvergence, Seoul National University, Seoul 08826, Republic of Korea

## Abstract

Eukaryotic cells repress global translation and selectively upregulate stress response proteins by altering multiple steps in gene expression. In this study, genome-wide transcriptome analysis of cellular adaptation to thermal stress was performed on the plant pathogenic fungus *Fusarium graminearum*. The results revealed that profound alterations in gene expression were required for heat shock responses in *F. graminearum*. Among these proteins, heat shock protein 90 (*Fg*Hsp90) was revealed to play a central role in heat shock stress responses in this fungus. *Fg*Hsp90 was highly expressed and exclusively localised to nuclei in response to heat stress. Moreover, our comprehensive functional characterisation of *Fg*Hsp90 provides clear genetic evidence supporting its crucial roles in the vegetative growth, reproduction, and virulence of *F. graminearum*. In particular, *Fg*Hsp90 performs multiple functions as a transcriptional regulator of conidiation. Our findings provide new insight into the mechanisms underlying adaptation to heat shock and the roles of Hsp90 in fungal development.

Heat stress affects a broad range of cellular processes that result in cell cycle arrest^1^, damage to membranes and cytoskeletal structures^2^, and accumulation of misfolded proteins^3^. To overcome these injuries, eukaryotic cells have evolved delicate heat shock response mechanisms that are primarily mediated by heat shock proteins (Hsps). Hsps are a family of proteins that are produced in response to thermal stress and are involved in various molecular functions, including molecular chaperone activity to assist with proper protein folding and disaggregation^4^, cell wall remodelling^5^, and maintenance of cell structures^2^. Although the number and nature of genes involved in the heat shock response vary between organisms, heat shock-related chaperones have been found to be rather conserved in eukaryotes^2^.

The Hsp90 is one of the most ubiquitous chaperones in eukaryotes, and its complex structure, interactions, and dynamic modifications have been comprehensively studied in humans and yeasts^6^. Hsp90, which constitutes 1–2% of the total protein in cytosol, is understood to be essential for the viability of *Saccharomyces cerevisiae*^7^, *Neurospora crassa*^8^, *Aspergillus fumigatus*^9^, and *Drosophila melanogaster*^10^. In *S. cerevisiae*, Hsp90 does not participate in the *de novo* folding of most proteins, but Hsp90 is specifically required for the proper folding of a subset of proteins that exhibits greater difficulty in reaching their native conformations^11^. Moreover, Hsp90 directly or indirectly controls the function of at least 10% of the entire proteome^11^ and physically interacts with calcineurin^12^. Hsp90 is expressed as two isoforms in human cells, a stress-induced isoform (Hsp90α) and a constitutively expressed isoform (Hsp90β), which perform distinct functions^13^. Whereas two Hsp90 orthologues (Hsp82 and Hsc82) have been reported in *S. cerevisiae*, only a single gene for Hsp90 has been identified in *Candida albicans* and in *A. fumigatus*^14^. Experiments inducing heterologous expression of human Hsp90 in *S. cerevisiae*^15^ and of *C. albicans* Hsp90 in *S. cerevisiae*^16^ confirmed that Hsp90s perform conserved biochemical functions in eukaryotes.

Genetic repression utilising conditional gene expression systems have been applied to study the biological roles of *HSP90*s in fungi including *S. cerevisiae*, *C. albicans*, *C. glabrata*, and *A. fumigatus* because of their lethal nature^9^,^17^,^18^,^19^. In *C. albicans*, repressing *HSP90* using a tetracycline-repressible promoter (*tetO*) results in attenuated virulence in mice^18^,^20^. Similarly, repressing *HSP90* using a thiamine-repressible promoter (*pthiA*) leads to a complete lack of growth *in vitro* and loss of virulence of *A. fumigatus*^9^,^19^. Moreover, repressing *AfHSP90* induces a defect in asexual sporulation, which is associated with downregulation of the conidiation-specific genes *BRLA*, *WETA*, and *ABAA*^9^. One study of *Af*Hsp90 indicated that observations derived from the model yeast *S. cerevisiae* cannot all be extrapolated to all other fungi. Moreover, little is known about the mechanistic roles of Hsp90 in filamentous fungi, and no Hsp90 orthologue has been functionally characterised in plant pathogenic fungi to date.

The ascomycete fungus *Fusarium graminearum* causes devastating *Fusarium* head blight in major cereal crops worldwide, leading to not only yield and quality losses but also contamination with harmful mycotoxins^21^. *F. graminearum* produces sexual spores (ascospores) within perithecia and asexual spores (conidia) on or within plant residues, including small grain stems and roots as well as maize stalks and ear pieces^22^. These spores are resistant to environmental stress conditions and are well suited for dispersal; thus, *F. graminearum* spores act as primary and secondary inocula^23^. Therefore, sexual and asexual reproduction by *F. graminearum* are important processes in the development of *Fusarium* head blight, and various genes and genetic pathways have been found to regulate these sporulation processes^22^,^24^.

The aim of this study was to further understand the heat shock response in the plant pathogenic fungus *F. graminearum*. As a first step, the RNA-seq-based transcriptome was analysed under optimal and elevated temperature conditions to identify genome-wide heat shock responses in this fungus. We found that the Hsp90 orthologue of *F. graminearum* plays the most active role in its response to thermal stress. Moreover, the important roles of Hsp90 in the life cycle of *F. graminearum* were investigated using various molecular genetic tools. Consequently, we comprehensively investigated not only the response of plant pathogenic fungi to heat stress but also the specific role of *Fg*Hsp90 in fungal development.

## Results

### Heat shock causes a profound modification of gene expression in *F. graminearum*

To dissect cellular responses to thermal stress in *F. graminearum*, we compared the transcriptomes of fungal cultures incubated under optimal (25 °C) and high temperature conditions (37 °C) for 15 min. Based on a threshold of reads per kilobase of exon per hundred million mapped reads (RPKHM) values (≥10) under all tested conditions, 9,638 out of 13,820 genes^24^ were selected for further analyses (Supplementary Dataset 1). Differentially expressed genes (DEGs) were identified as genes displaying a greater than two-fold change in transcript levels. Compared with optimal growth conditions (25 °C), heat shock conditions induced the differential expression of 5,983 genes (1,975 genes were upregulated and 4,008 genes were downregulated). These results emphasise a profound modification of gene expression, such that ~43% of the total genes were differentially expressed in this fungus.

We evaluated the transcript levels of genes possibly related to thermal stress responses based on GO terms (GO: 0009266, response to temperature stimulus; GO: 0009408, response to heat; GO: 0034605, cellular response to heat) and found only seven DEGs among 44 genes (Table 1). Notably, a ubiquitous chaperone, heat shock protein 90 (*Fg*Hsp90, FGSG_02014), was revealed to be most highly induced under heat stress conditions. In addition, the co-chaperone with Hsp90 (FGSG_00766, *STI1*) and one ubiquitin gene (FGSG_08768, *UBI4*) were highly expressed under high temperature conditions. The transcript levels were further confirmed using quantitative real-time (qRT)-PCR.

### Transcription of *FgHSP90* is induced in response to heat stress and during sexual and asexual development

**Figure 1:**
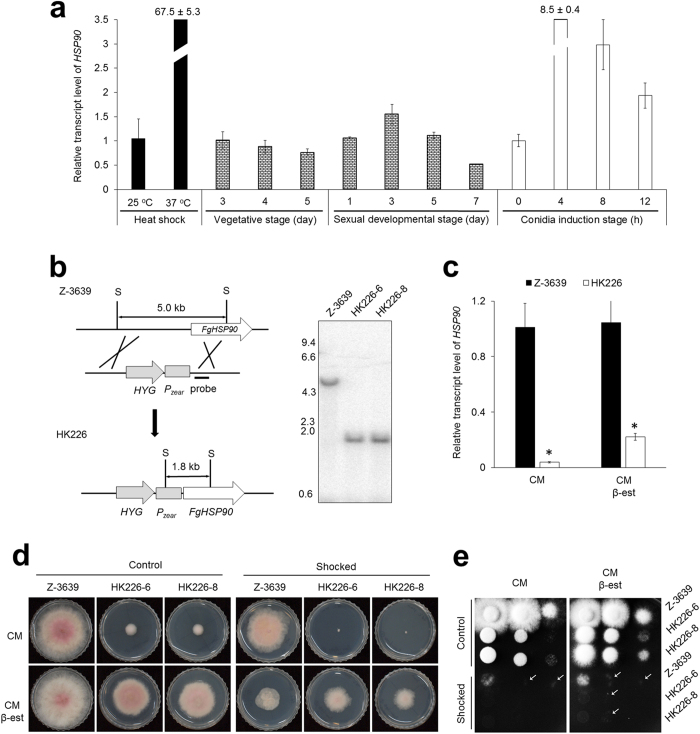
Characterisation of the mutant containing the *FgHSP90* gene under the control of a ZEA-inducible promoter (*P*_*zear*_). (**a**) Expression profile of the *FgHSP90* gene in the *F. graminearum* wild-type strain Z-3639 under heat shock conditions and during development. Transcript levels were analysed via qRT-PCR after heat shock treatment at 37 °C for 15 min, during the vegetative and sexual induction stages on carrot agar, and during conidia induction on YMA medium. During the vegetative and sexual induction stages, the transcript level of *FgHSP90* at the 3-day vegetative stage was arbitrarily set to 1, and this value was used for comparison to other periods. During the conidial induction stage, the expression of *FgHSP90* at 0 h was arbitrarily set to 1, and this value was used for comparison to other periods. (**b**) An overview of the strategy for promoter replacement. Southern blot hybridisation assay confirmed that the Z-3639 strain (lane 1) migrated as a 5.0 kb fragment, but the positively mutated strains HK226-6 and HK226-8 (lanes 2 and 3, respectively) migrated as 1.8-kb bands. (**c**) Confirmation of chemical complementation. The transcript level of *FgHSP90* in the Z-3639 and HK226 strains was analysed by qRT-PCR. The relative transcript levels in the wild-type strain were arbitrarily set to 1. (**d**) Mycelial growth of *F. graminearum* strains under heat shock conditions. Both wild-type HK226 strains were incubation for 30 min at 48 °C for heat shock stress in the absence and presence of β-est. The images were captured at 3 days after inoculation. (**e**) Serial dilutions of all strains were point-inoculated onto CM with and without β-est after 30 min heat shock treatment at 48 °C. Arrowheads indicate mycelial growth.

qRT-PCR confirmed that the transcript level of *FgHSP90* was dramatically increased in response to elevated temperature (Fig. 1a). Further, the transcript profiles of *FgHSP90* were examined throughout vegetative growth as well as in the stages of asexual and sexual development to elucidate the roles of *FgHSP90* in the life cycle of *F. graminearum*. Although the transcript levels of *FgHSP90* were constitutively maintained during the vegetative growth stages, the *FgHSP90* transcript levels were significantly increased 3 days after sexual induction, followed by a decrease 3 days later (Fig. 1a). Notably, the *FgHSP90* transcript level peaked 4 h after conidial induction and gradually decreased thereafter (Fig. 1a). Taken together, these results indicate that *Fg*Hsp90 is closely involved in sexual and asexual developmental processes as well as heat shock responses in *F. graminearum*.

### Molecular characterisation of *Fg*Hsp90

Hsp90 proteins in eukaryotes range in size from 588 to 854 amino acids^13^. BLASTp searches for both Hsp82 and Hsc82 from *S. cerevisiae* in the *F. graminearum* genome^24^ identified the FGSG_02014 locus encoding 700 amino acids (73% and 74% identity with *Sc*Hsp82 and *Sc*Hsc82, respectively). The encoded protein harboured two important domains (IPR004358 and IPR001404) similar to those in both *Sc*Hsp82 and *Sc*Hsc82 (Supplementary Fig. S1a). Our result was in agreement with the previously characterised *A. fumigatus* orthologue *Af*Hsp90, which consists of 706 amino acids and displays approximately 75% identity to the yeast Hsp90 orthologues^14^. Whereas human and *S. cerevisiae* carry two Hsp90 homologues, a single Hsp90 isoform is carried by other fungi including *C. albicans*, *Podospora anserina*, and *A. fumigatus*^13^,^14^,^25^. Based on these results, we designated the protein encoded by FGSG_02014 and referred to this protein as *Fg*Hsp90, a unique orthologue of Hsp90 in *F. graminearum*.

*Fg*Hsp90 contains a nuclear localisation sequence (NLS; 246-KPKTKK-251) at its N-terminus (Supplementary Fig. S1a). Similar to other Hsp90 isoforms in eukaryotes, two evolutionary conserved motifs, NKEIFL and MEEVD, were present in *Fg*Hsp90 (Supplementary Fig. S1b). The MEEVD motif is known to interact with tetratricopeptide repeat domains of the co-chaperone Hsp90-Hsp70 organizing protein (Hop, also known as p60 or Sti1) in humans and yeast and is therefore important for the transfer of client proteins from Hsp70 to Hsp90^26^. Phylogenetic analyses of Hsp90 orthologues showed that Hsp90s of filamentous fungi were clustered into a separate group relative to Hsp90 from other organisms (Supplementary Fig. S1c).

### *FgHSP90* is an essential gene in *F. graminearum*

As a first step towards investigating the genetic function of *FgHSP90* in *F. graminearum*, we attempted to delete this gene using the homologous recombination method^27^. Because more than three repeated attempts to delete *FgHSP90* had failed, we concluded that *FgHSP90* is an essential gene in *F. graminearum*, as in yeasts and other filamentous fungi^7^,^8^,^9^,^19^. Next, we utilised a conditional gene expression system to repress *FgHSP90*. The zearalenone (ZEA)-inducible promoter (*P*_*zear*_) is the only available conditional gene expression system for *F. graminearum*^28^. Furthermore, the potential for the inexpensive reagent β-estradiol (β-est) to substitute for ZEA in this conditional gene expression system has been demonstrated, thereby facilitating the utilisation of this system in high-throughput studies^29^.

We generated HK226 strains in which the promoter of the *FgHSP90* gene was replaced with *P*_*zear*_ and confirmed the preciseness of this genetic manipulation using Southern blot analysis (Fig. 1b). HK226 mutant strains displayed dramatically reduced transcript levels of *FgHSP90* (Fig. 1c) and severely defective mycelial growth compared to the wild-type strain (Table 2 and Fig. 1d). Adding β-est partially restored radial growth and reduced the transcript level of *FgHSP90*. These results indicated that the *P*_*zear*_ system is applicable to this study. There was no apparent difference in mycelial morphology between the HK226 mutant and wild-type strains (data not shown).

To examine the requirement of *FgHSP90* for heat shock adaptation, heat shock (48 °C for 30 min) was applied to both the HK226 mutant and wild-type strains. Most HK226 mutant strains were inviable after heat shock treatment, whereas the wild-type strain exhibited slightly delayed radial growth (Fig. 1d). When β-est was applied, the two heat shock-treated strains grew similarly. The germination rate of serial dilution assays confirmed that most of HK226 mutant conidia were blocked to germinate after heat shock treatment, whereas the growth rate of wild-type strain were only retarded (Fig. 1e). Adding β-est partly restored the growth and viability of HK226 mutants. We also examined the sensitivity of the HK226 mutant strains to various stress conditions, including osmotic and oxidative stresses, cell wall-damaging agent exposure, fungicide exposure, and altered pH. None of these stress conditions altered the growth of HK226 mutant strains (Supplementary Fig. S2).

### *FgHSP90* is required for conidiation in *F. graminearum*

**Figure 2:**
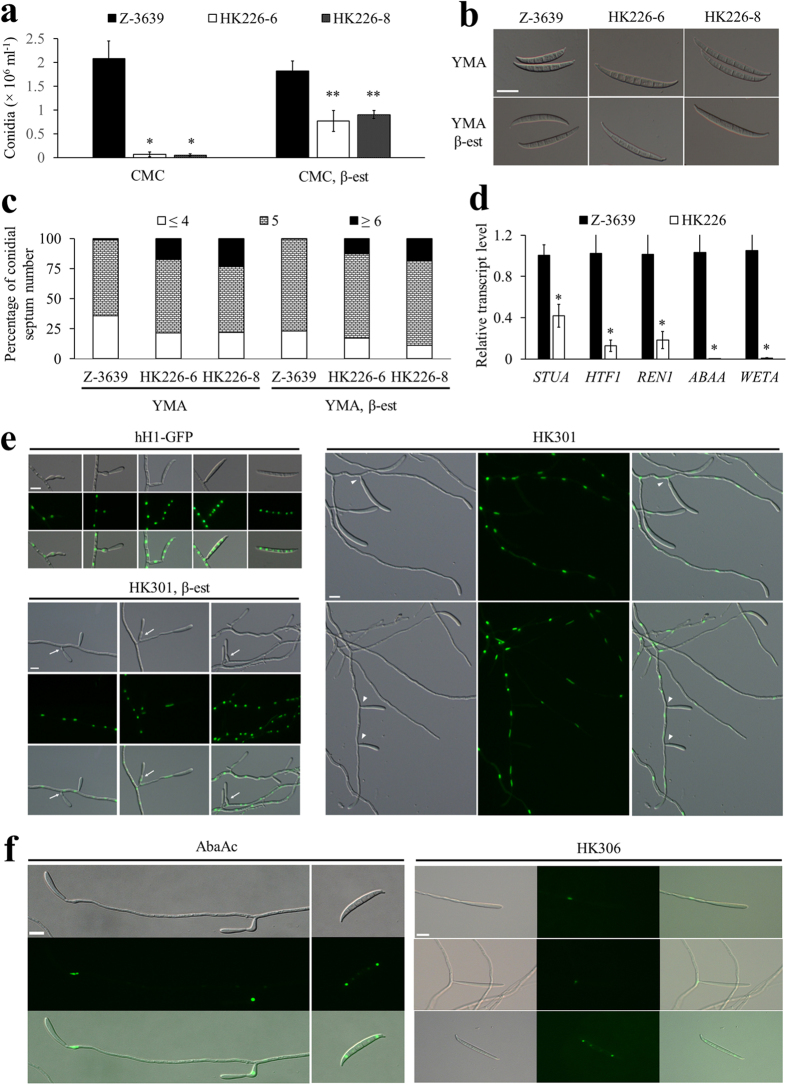
Conidiation in the repression of *FgHSP90* mutants. (**a**) Conidial production. The number of conidia was counted after 5 days of incubation in CMC in the absence or presence of β-est. The values were generated based on three biological replicates. (**b**) Conidial morphology. Conidia were induced on YMA in the absence or presence of β-est and subsequently observed by DIC microscopy. Scale bar = 20 μm. (**c**) Percentage of conidial septa. One hundred conidia induced on YMA were assessed for each strain with three biological replicates. (**d**) Relative transcript levels of genes related to conidiation. Total RNA was extracted from the wild-type and HK226 strains cultured in CM for 48 h and then subjected to asexual induction in CMC for 6 h. The relative transcript levels of each gene in the wild-type strain were arbitrarily set to 1. (**e**) Morphology of conidiophores in *F. graminearum* strains. Images were captured 1 to 3 days after conidial induction on CMC in the absence or presence of β-est. Arrows and arrowheads indicate phialides and conidia directly produced from hyphae, respectively. Scale bar = 10 μm. (**f**) Cellular localisation of AbaA-Gfp in the strain with wild-type allele of *FgHSP90* (left) and in the *FgHSP90*-repressed strain (right). Scale bar = 10 μm.

Genetic repression of *FgHSP90* resulted in severe defects in asexual sporulation. First, conidial production by the HK226 strains was markedly reduced compared to that by the wild-type strain, but supplementation with β-est partially restored defective conidiation by the HK226 strains (Fig. 2a). Furthermore, the conidial morphologies of the HK226 strains were significantly altered. The conidia of the HK226 strains tended to be longer and thinner than those of the wild-type strain. HK226 conidia had more septa than wild-type conidia; this difference was observed due to the occurrence of conidia with 6 ~8 septa in the HK226 strains (Table 2 and Fig. 2b,c).

To gain further insight into the role of *FgHSP90* in the asexual sporulation process in *F. graminearum*, we observed conidiophore morphogenesis at the microscopic level. To visualise nuclei, the HK301 strains (*P*_*zear*_*-FgHSP90 hH1-GFP-HYG*) were generated by outcrossing the mat1g and HK226 strains (Supplementary Table S1). The hH1-GFP strain, carrying the wild-type allele of *FgHSP90*, initially produced phialides from the hyphae, and conidia were subsequently formed from mature phialides (Fig. 2e). In addition, conidia were often directly produced from hyphae. However, genetic repression of *FgHSP90* abolished phialide formation, and consequently, most conidia were directly produced from hyphae or at the hyphal tips (arrowheads in Fig. 2e). Phialide production by the HK301 mutant strains was partly restored by supplementing the cultures with β-est (arrows in Fig. 2e).

We further investigated the possible link between *FgHSP90* and representative conidiation-related genes in the wild-type and HK226 strains. The transcript levels of five genes, *STUA*^30^, *HTF1*^31^, *REN1*^32^, *ABAA*^33^, and *WETA*^34^, were significantly decreased in the HK226 mutant strains compared to the wild-type strain (Fig. 2d). Although the expression levels of *ABAA* and *WETA*, transcription factors specifically involved in conidiogenesis in *F. graminearum*^33^,^34^, were generally undetectable in the HK226 mutant strains (Fig. 2d), the HK305 (Δ*abaA P*_*zear*_*-FgHSP90*) strains (Supplementary Table S1) could not produce conidia in either YMA or CMC medium (data not shown). To further confirm the necessity of AbaA for conidiation, we next visualised the localisation of this protein during the conidiation stage. As noted in a previous report^33^, AbaA-Gfp was highly accumulated in the phialides and in the nuclei of terminal cells of maturing conidia in AbaAc strains (Fig. 2f). The expression of AbaA-Gfp in the HK306 (*ABAA-GFP P*_*zear*_*-FgHSP90*) strains showed an identical pattern (Fig. 2f). The conidial germination rates of the HK226 mutant strains were similar to those of the wild-type strain, whereas *HSP90*-repressed *A. fumigatus* mutant strains showed retarded conidial germination^9^. These findings suggest that *FgHSP90* is dispensable for germination in *F. graminearum*.

### *FgHSP90* is involved in virulence and sexual development

**Figure 3:**
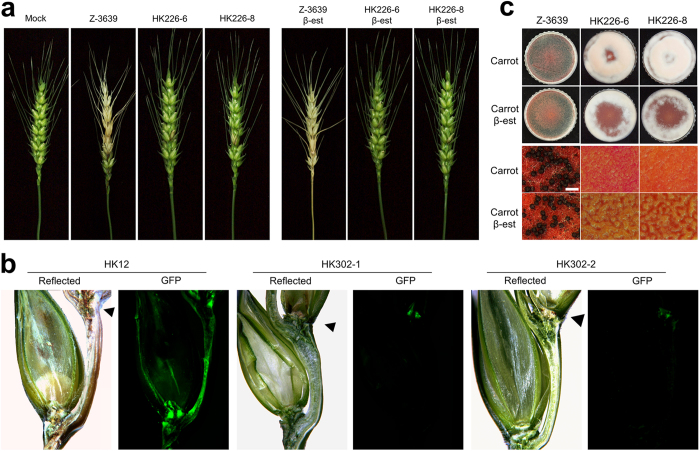
Virulence and sexual development. (**a**) Virulence on wheat heads. The centre spikelet of each wheat head was injected with 10 μl of a conidial suspension. Images were captured 21 days after inoculation. (**b**) Micrographs of manually generated sections after infection of wheat. Wheat spikelets were inoculated with conidial suspensions from strains expressing Gfp in the cytoplasm (HK12 and HK302). Infected wheat heads were longitudinally dissected 6 days after inoculation and examined under a fluorescence microscope. Gfp fluorescence represents hyphae spreading from the inoculation points. Arrowheads mark the inoculated spikelets. Reflected, reflected light. (**c**) Sexual development. A five-day-old culture in carrot agar was mock-fertilised to induce sexual production and incubated for an additional 7 days. The upper and below panels show photographs of self-fertility on a whole carrot agar plate and photographs captured using a dissecting microscope, respectively. Scale bar = 500 μm.

To determine the role of *FgHSP90* in pathogenicity, we performed a virulence assay on flowering wheat heads by point inoculation. The wild-type strain caused typical head blight symptoms 21 days after inoculation as expected, whereas the HK226 strains were unable to spread from the inoculated spikelet to adjacent spikelets on the heads (Table 2 and Fig. 3a). In contrast to its effects on the growth rate and conidiation, supplementation with β-est did not restore the virulence of the HK226 mutant strains.

To further visualise the movement of mycelia during infection of wheat heads at the microscopic level, we generated HK302 (*P*_*zear*_*-FgHSP90 GFP-HYG*) strains by outcrossing the KM19 (Δ*mat1::GEN GFP-HYG*, constitutively expressing green fluorescent protein [Gfp]) and HK226 strains (Supplementary Table S1). The HK12 strain, carrying the wild-type allele of *FgHSP90*, spread hyphae to the adjacent spikelet through its rachis node (Fig. 3b). The hyphae of HK302 strains were observed only at the inoculated points of the rachis, and these strains failed to colonise the injected spikelet and spread to the adjacent spikelet (Fig. 3b).

We next examined the role of *FgHSP90* in sexual development. The wild-type strain began to produce detectable perithecial initials 3 days after sexual induction, and mature perithecia were subsequently produced after an additional 3 or 4 days of incubation (Fig. 3c). In contrast to the wild-type strain, repressing *FgHSP90* completely blocked the production of perithecia even in the presence of β-est (Fig. 3c).

### The subcellular localisation pattern of *Fg*Hsp90 reveals its multiple functions during the conidiation stage

To examine the subcellular localisation of *Fg*Hsp90 during the developmental stages of *F. graminearum*, the HK227 strains expressing the *Fg*Hsp90-Gfp fusion protein were generated (Supplementary Fig. S3). Dozens of transformants containing a single copy of *FgHSP90-GFP* were obtained, and nuclear Gfp fluorescent signals were observed in these strains. For confirmation of the nuclear localisation of *Fg*Hsp90, HK303 (*FgHSP90-GFP-HYG hH1-RFP-HYG*) strains were generated by outcrossing the HK227 strain and the mat1r strain, which carries a gene encoding red fluorescent protein (Rfp) fused to the histone H1 protein in a *MAT1-1* deletion background.

**Figure 4:**
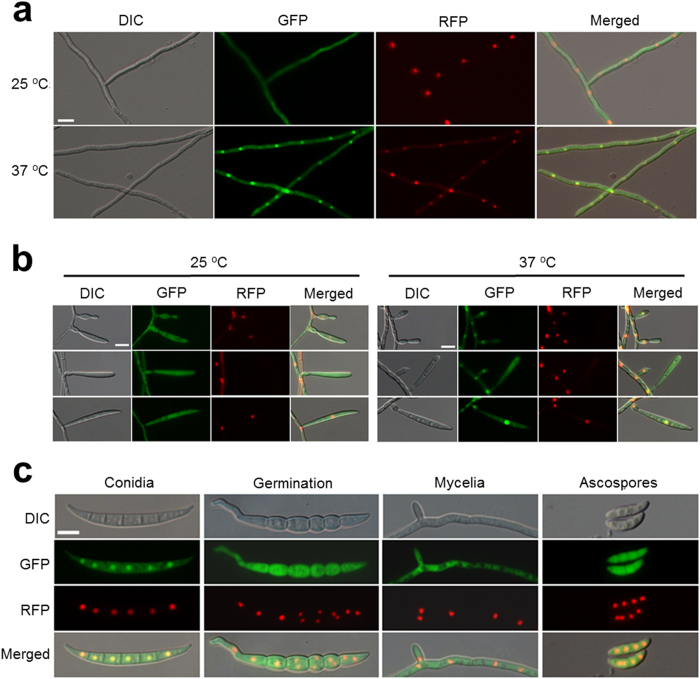
Localisation of *Fg*Hsp90 in *F. graminearum*. Representative images showing co-localisation of *Fg*Hsp90 fused to Gfp with histone H1 fused to Rfp. (**a**) *Fg*Hsp90 translocated to nuclei from the cytosol after heat shock treatment at 37 °C for 1 h. Scale bar = 10 μm. (**b**) Localisation of *Fg*Hsp90 during conidiogenesis in CMC under optimal conditions (left) and heat shock treatment (37 °C for 1 h) (right). Scale bar = 10 μm. (**c**) A strain containing *FgHSP90-GFP* and *hH1-RFP* was grown on YMA (conidia), CM (germination and mycelia), or carrot agar medium (ascospores) for microscopic observation. DIC, differential interference contrast. Scale bar = 10 μm.

We next investigated the localisation patterns of *Fg*Hsp90 in hyphae and conidiophores under optimal and heat shock conditions. Interestingly, a distinct shift in Gfp signals from the cytosol to the nuclei was observed after heat shock treatment at 37 °C (Fig. 4a,b). Similarly, an in-depth examination of Hsp90 localisation during conidiogenesis showed that *Fg*Hsp90 was highly accumulated in the nuclei of mature phialides and conidia (Fig. 4b); this expression pattern corresponds to the functions of *Fg*Hsp90 in the late stages of conidiation. *Fg*Hsp90 was evenly distributed throughout the cytoplasm after conidial germination (Fig. 4c). Distinct nuclear localisation of *Fg*Hsp90 was not observed in ascospores (sexually produced spores) (Fig. 4c). However, germination rate and viability of conidia and ascospores under heat shock stress were indistinguishable (Fig. S4).

## Discussion

In this study, the heat shock response in the plant pathogenic fungus *F. graminearum* was investigated for the first time using transcriptome analysis. The heat shock response accompanied a dramatic alteration in the gene expression profile in *F. graminearum*. In particular, we identified the heat shock protein *Fg*Hsp90, which appeared to play the prominent roles in response to thermal stress. In-depth functional analyses revealed that *Fg*Hsp90 also performs crucial functions in *F. graminearum* during various developmental stages including vegetative growth, asexual and sexual reproduction, and virulence. Moreover, subcellular localisation patterns supported that *Fg*Hsp90 performs multiple functions as a transcriptional regulator as well as a chaperone during conidiation. Taken together, the results of our study shed light into the roles of the heat shock protein Hsp90 in fungal development.

Hsp90 is a conserved molecular chaperone that functions in the refolding of denatured and/or aggregated proteins generated by high temperature stress^6^. We found that *Fg*Hsp90 plays the most active role in the response to heat stress in *F. graminearum* compared to other genes related to responses to temperature (Table 1). Moreover, the orthologue for the Hsp90 co-chaperone Sti1 was also highly expressed under thermal stress conditions. StiA (a homologue of Sti1 in *A. fumigatus*) is not required for the physical interaction between Hsp70 and Hsp90 but does play distinct roles in the regulation of Hsp90 by inhibiting ATPase activity^26^,^35^. Taken together, this evidence indicates that *Fg*Hsp90 and related regulatory pathways play key roles in thermotolerance in *F. graminearum*.

Our study confirmed that *FgHSP90* is an essential gene that performs diverse functions in fungal development and virulence. In *C. albicans*, *Ca*Hsp90 functions as a regulator of morphology by repressing Ras/PKA pathway^20^, and *Ca*Hsp90 is required for virulence^18^. *Af*Hsp90 of *A. fumigatus* is involved in spore viability, hyphal growth, conidiation and virulence^9^,^19^. Similarly, repression of *FgHSP90* resulted in pleiotropic phenotypic defects including deficiencies in morphogenesis and virulence, and these observations support the conserved roles of Hsp90 among fungi. However, the involvement of Hsp90 in sexual development was first reported in *F. graminearum*. In addition, although *AfHSP90* plays a crucial role in the regulation of cell wall integrity pathways in *A. fumigatus*^9^, *FgHSP90* repression led to wild-type phenotypes under various stress conditions including exposure to cell wall-damaging agents. Taken together, these results indicate that Hsp90s play highly conserved roles in the fungal kingdom but that some differences in their function between species might be attributed to different physiologies among fungi and variations in regulatory mechanisms of Hsp90 at the post-translational level.

*AfHSP90* repression induces a defect in asexual sporulation, affecting both the production and pigmentation of conidia, and this defect is associated with downregulation of the conidiation-specific genes *BRLA*, *WETA* and *ABAA*^9^. Similarly, our in-depth examination of Hsp90 function in *F. graminearum* verified its specific role in asexual sporulation. *FgHSP90* was highly expressed during conidiogenesis and participated in the formation of conidiogenous cells, referred to as phialides, by upregulating conidiation-specific genes (*STUA*, *HTF1*, *REN1*, *ABAA*, and *WETA*). Moreover, *Fg*Hsp90 was highly accumulated in nuclei at the late stage of conidiophores and conidia, and this localisation pattern suggests a role of *Fg*Hsp90 as a transcriptional regulator specifically involved in conidiation. Specific nuclear localisation of Hsp90 orthologues during conidiation has not been reported in other organisms, although stress conditions commonly induce nuclear localisation of Hsp90^9^.

In conclusion, this study revealed the key roles of *Fg*Hsp90 in heat shock responses and fungal development via genome-wide transcriptome analysis in the plant pathogenic fungus *F. graminearum*. Comprehensive functional characterisation of *Fg*Hsp90 demonstrated that *Fg*Hsp90 performs crucial functions in vegetative growth, reproduction, and virulence. We also produced evidence that *Fg*Hsp90 performs multiple functions as a transcriptional regulator as well as a chaperone for conidiation. The results of this study provide new insight into the mechanistic roles of Hsp90 in fungal development and virulence.

## Methods

### Fungal strains and media

The *F. graminearum* wild-type strain Z-3639^36^ and the mutants used in this study are listed in Supplementary Table S1. Standard laboratory methods and culture media for *Fusarium* species were used^37^. For fungal sporulation, conidia of all strains were induced on yeast malt agar (YMA)^38^ or in carboxymethyl cellulose (CMC) medium^39^. The growing temperature of fungal strains was set at 25 °C unless otherwise specified. The wild-type and transgenic strains were stored as mycelia and conidia in 30% glycerol at −80 °C.

### Nucleic acid manipulation, primers, and PCR conditions

The genomic DNA was extracted following the standard protocol^37^. Restriction endonuclease digestion, agarose gel electrophoresis, gel blotting, and DNA blot hybridisation were performed in accordance with standard techniques^40^. The PCR primers (Supplementary Table S2) used in this study were synthesised by an oligonucleotide synthesis facility (Bionics, Seoul, Korea).

### Genetic modifications

To replace the *FgHSP90* promoter with *P*_*zear*_, the hygromycin resistance gene cassette (*HYG*)*-P*_*zear*_ was amplified from the *P*_*zear*_*-GzmetE* strain^28^ using the HYG-F1 and zear-r2 primers, and the 5′ and 3′ flanking regions of the *FgHSP90* gene were amplified from Z-3639 using the primers FgHSP90-5F pzear/FgHSP90-5R pzear and FgHSP90-3F pzear/FgHSP90-3R pzear, respectively. The resulting three fragments were fused according to the double-joint (DJ) PCR method^27^, and the final construct was amplified using the primers FgHSP90-5N pzear/FgHSP90-3N pzear. To induce *P*_*zear*_ replacement, 30 μM β-est was added to the medium during the regeneration, overlay, and mutant selection processes^28^.

To generate *GFP*-tagged *Fg*Hsp90 strains, the *GFP-HYG* fragment was amplified from the pIGPAPA plasmid^41^ using the primers pIGPAPA-sGFP/HYG-F1. The 5′ and 3′ flanking regions of the *FgHSP90* gene were amplified from Z-3639 using the primers FgHSP90-5F GFP/FgHSP90-5R GFP and FgHSP90-3F GFP/FgHSP90-3R GFP, respectively. After fusion PCR, the resulting PCR product was used as a template together with the primers FgHSP90-gfpF and FgHSP90-gfpR to produce the final construct. Subsequently, the final PCR products were transformed into the Z-3639 strain.

### Conidial production

After each strain was incubated in 50 ml of complete medium (CM) for 72 h at 25 °C on a rotary shaker (150 rpm), mycelia of each strain were harvested and washed twice with distilled water. To induce conidiation, 72 h-old mycelia were spread on YMA and incubated for 48 h at 25 °C under near-UV light (wavelength: 365 nm, HKiv Import & Export Co., Ltd., Xiamen, China). Conidia were collected using distilled water, filtered through cheesecloth, washed, and resuspended in distilled water. After inoculating a 1-ml conidial suspension (1 × 10^6^ conidia/ml) of each strain in 50 ml of CMC and incubating this culture for 5 days at 25 °C on a rotary shaker (150 rpm), the number of conidia produced was counted using a haemocytometer (Superior, Marienfeld, Germany) to measure conidial production.

### Sexual development and virulence tests

Mycelia grown on carrot agar for 5 days were mock-fertilised with sterile 2.5% Tween 60 solution to induce sexual reproduction as previously described^37^. After sexual induction, the fertilised cultures were incubated for 7 days under near-UV light (HKiv Import & Export Co., Ltd.) at 25 °C.

A virulence test of the fungal strains was performed using the wheat cultivar Eunpamil as previously described^42^. Briefly, 10 μl of a conidial suspension (1 × 10^5^ conidia/ml) obtained from each strain was point-inoculated onto a spikelet of the wheat head at early anthesis. Inoculated plants were incubated in a humidified chamber for 3 days and subsequently transferred to a greenhouse. After 21 days, the number of spikelets showing disease symptoms was counted.

### Microscopic observation

Microscopic observation was performed using a DE/Axio Imager A1 microscope (Carl Zeiss, Oberkochen, Germany) equipped with the filter set 38HE (excitation 470/40; emission 525/50) for Gfp and the filter set 15 (excitation 546/12; emission 590) for Rfp.

Wheat heads inoculated with the Gfp-tagged strains were sampled 6 days after inoculation. Freehand longitudinal sections across the centre of the spikelets were prepared using a clean scalpel^43^. Sectioned wheat heads were observed under reflected light and Gfp-fluorescent light (470-nm excitation and 525-nm emission wavelength filters) using a SteREO Lumar V12 microscope (Carl Zeiss).

### qRT-PCR

Mycelia were harvested via filtration through one or two layers of Miracloth, washed with water, frozen in liquid nitrogen, lyophilised, and ground in a mortar and pestle prior to RNA extraction using the Easy-Spin total extraction kit (iNtRON Biotech, Seongnam, Korea). First-strand cDNA was synthesised with the SuperScript III First-Strand Synthesis System (Invitrogen, Carlsbad, CA, USA) using oligo(dT)_20_ according to the manufacturer′s recommendations. qRT-PCR was performed using iQ SYBR Green Master Mix (Bio-Rad, Hercules, CA, USA) and a 7500 real-time PCR system (Applied Biosystems, Foster City, CA, USA). The endogenous housekeeping gene ubiquitin C-terminal hydrolase (*UBH*; FGSG_01231) was used for normalisation^44^. The PCR assays were repeated three times with two biological replicates. The transcript level relative to that of the housekeeping gene was expressed as 2^−ΔΔCT ^45.

### RNA-seq and bioinformatic analysis

Conidia were inoculated in CM for 12 h at 25 °C and then further incubated at 37 °C for 15 min for heat shock treatment. Total RNA was extracted using the Easy-Spin total extraction kit (iNtRON Biotech) as described above. RNA-seq libraries were created using the Illumina TruSeq RNA sample preparation kit strictly according to the standard low-throughput protocol. Sequencing was performed using an Illumina HiSeq 2000 instrument and the reagents provided in the Illumina TruSeq paired-end (PE) Cluster kit v3-cBot-HS and the TruSeq SBS kit v3-HS (200 cycles). Each experiment was repeated three times, and mean values were used for bioinformatic analyses.

The data have been deposited in NCBI’s Gene Expression Omnibus^46^ and are accessible at GEO Series accession number GSE78885 (http://www.ncbi.nlm.nih.gov/geo/query/acc.cgi?acc = GSE78885). The genome-wide transcript levels of genes were quantified as RPKHM^47^. When the RPKHM value was 0, it was changed to 1 to calculate the fold change of the transcript level.

## Additional Information

**How to cite this article**: Bui, D.-C. *et al*. Heat shock protein 90 is required for sexual and asexual development, virulence, and heat shock response in *Fusarium graminearum*. *Sci. Rep.* **6**, 28154; doi: 10.1038/srep28154 (2016).

## Supplementary Material

Supplementary Information

Supplementary Dataset 1

## Figures and Tables

**Table 1:** Transcript levels of genes involved in responses to temperature.

Locus	MIPS annotation	GO term	Mean RPKHM		qRT-PCR
			25 °C	37 °C	
FGSG_02014	probable heat shock protein 90	GO:0009266, GO:0009408	18,507	885,938	67.5 ± 5.3
FGSG_00766	related to stress-induced protein *STI1*	GO:0009266, GO:0009408	17,184	318,411	38.2 ± 2.0
FGSG_10816	related to aquaporin	GO:0009266	12	196	54.3 ± 8.4
FGSG_03247	probable *GUT1*-glycerol kinase	GO:0009266	40	523	42.7 ± 2.5
FGSG_08768	probable *UBI4*-ubiquitin	GO:0009266, GO:0009408, GO:0034605	49,752	514,937	23.9 ± 0.8
FGSG_16768	related to Krüppel protein	GO:0009266	912	6454	23.5 ± 4.3
FGSG_02316	related to multidrug resistance protein	GO:0009266, GO:0009408	451	1078	5.9 ± 0.5

RPKHM, reads per kilobase of exon per hundred million mapped sequence reads^47^; 25 °C and 37 °C, transcript levels of genes expressed at 25 °C and 37 °C, respectively; qRT-PCR, the fold-change in gene expression at 37 °C relative to that at 25 °C confirmed by qRT-PCR analysis.

**Table 2:** Vegetative growth, conidial morphology, and virulence of the *FgHSP90*-repressed mutants.

Strain	Radial growth (mm)		Conidial morphology						Virulence (disease index)	
	CM	CM β-est	YMA			YMA β-est			-	β-est
			Length (μm)	Width (μm)	No. of septa	Length (μm)	Width (μm)	No. of septa		
Z-3639	63.5	48.6	53.0	5.8	4.5	54.5	5.8	4.7	11.5	9.1
HK226-6	12.6*	34.6*	62.6*	5.5*	4.9*	60.6*	5.7	5.0*	0.7*	0.6*
HK226-8	10.8*	33.1*	65.5*	5.6*	4.9*	64.8*	6.1	5.0*	0.6*	0.9*

Radial growth was measured 3 days after inoculation. Conidia were harvested from a 1-day-old culture in yeast malt agar (YMA). The disease index (number of diseased spikelets per wheat head) of the strains was measured 21 days after inoculation. CM, complete medium; β-est, β-estradiol.

^*^data differed significantly (*P* < 0.05) based on Tukey’s test within each column; Z-3639, *F. graminearum* wild-type strain; HK226, *F. graminearum FgHSP90*-repressed strains.
