# Supplementary material for: Heat shock protein 90 is required for sexual and asexual development, virulence, and heat shock response in Fusarium graminearum
Source: Sci Rep. 2016 Jun 16;6:28154. doi: 10.1038/srep28154 (PMC4910114; doi:10.1038/srep28154)
Supplement: Supplementary Information [file srep28154-s1.doc]

**Supplementary information**

**Heat shock protein 90 is required for sexual and asexual development, virulence, and heat shock response in *Fusarium graminearum***

Duc-Cuong Bui^1^, Yoonji Lee^1^, Jae Yun Lim^1^, Minmin Fu^1^, Jin-Cheol Kim^2^, Gyung Ja Choi^3^, Hokyoung Son^4^ & Yin-Won Lee^1^

^1^Department of Agricultural Biotechnology, Seoul National University, Seoul 08826, Republic of Korea. ^2^Division of Applied Bioscience and Biotechnology, Institute of Environmentally Friendly Agriculture, College of Agriculture and Life Sciences, Chonnam National University, Gwangju 61186, Republic of Korea. ^3^Eco-friendly New Materials Research Group, Research Centre for Biobased Chemistry, Division of Convergence Chemistry, Korea Research Institute of Chemical Technology, Daejeon 34114, Republic of Korea. ^4^Centre for Food and Bioconvergence, Seoul National University, Seoul 08826, Republic of Korea.

Correspondence and requests for materials should be addressed to H.S. (email: hogongi7@snu.ac.kr) or Y.-W.L. (email: lee2443@snu.ac.kr)

| **Strain** | **Genotype** | **Reference, source, or parent strains** |
| --- | --- | --- |
| Z-3639 | *Fusarium graminearum* wild-type | [^1^](#_ENREF_1) |
| hH1-GFP | *hH1::hH1-GFP-HYG* | [^2^](#_ENREF_2) |
| *P_zear_-GzmetE* | *GzMETE::HYG-P_zear_-GzmetE* | [^3^](#_ENREF_3) |
| mat1g | Δ*mat1-1::GEN hH1::hH1-GFP-HYG* | [^2^](#_ENREF_2) |
| mat1r | Δ*mat1-1::GEN hH1::hH1-RFP-HYG* | [^4^](#_ENREF_4) |
| HK12 | *GFP-HYG* (GFP constitutive expresser) | [^5^](#_ENREF_5) |
| Δ*mat1* | Δ*mat1-1::GEN* | [^6^](#_ENREF_6) |
| Δ*mat2* | Δ*mat1-2::GFP-HYG* | [^6^](#_ENREF_6) |
| KM19 | Δ*mat1-1-1::GEN GFP-HYG* | [^7^](#_ENREF_7) |
| HK226 | *FgHSP90::HYG-P_zear_-FgHSP90* | This study |
| HK227 | *FgHSP90::FgHSP90-GFP-HYG* | This study |
| HK301 | *FgHSP90::HYG-P_zear_-FgHSP90 hH1::hH1-GFP-HYG* | mat1g×HK226 |
| HK302 | *FgHSP90::HYG-P_zear_-FgHSP90 GFP-HYG* | KM19×HK226 |
| HK303 | *FgHSP90::FgHSP90-GFP-HYG hH1::hH1-RFP-HYG* | mat1r × HK227 |
| HK167 | Δ*mat1-2::GFP-HYG* Δ*abaA::GEN* | This study |
| AbaAc | Δ*abaA::ABAA-GFP-HYG* | [^8^](#_ENREF_8) |
| HK304 | Δ*mat1-1::GEN* Δ*abaA::ABAA-GFP-HYG* | Δ*mat1*× AbaAc |
| HK305 | *FgHSP90::HYG-P_zear_-FgHSP90* Δ*abaA::GEN* | HK167 × HK226 |
| HK306 | *FgHSP90::HYG-P_zear_-FgHSP90* Δ*abaA::ABAA-GFP-HYG* | HK304 × HK226 |

**Table S1. *F. graminearum* strains used in this study.**

| **Primer** | **Sequence (5’→3’)** | **Description** |
| --- | --- | --- |
| FgHSP90-5F pzear | CCAAGTCAATAACTGCGTCCTGTTC | Forward and reverse primers for amplification of 5′-flanking region of *FgHSP90* with tail for the hygromycin resistance gene cassette fusion |
| FgHSP90-5R pzear | tccactagctccagccaagccAGCCCACCTAGCGCTGCCTA |
| FgHSP90-3F pzear | gagagaacgaaagtaaccatgCTCTCACGCTTTTTGCACATTCTCT | Forward and reverse primers for amplification of 3′-flanking region of *FgHSP90* with tail for the geneticin resistance gene cassette fusion |
| FgHSP90-3R pzear | GCGTACTCGTCAATGGGGTCAA |
| FgHSP90-5N pzear | TCATTGTGGTGGGAGAGGTGGTAG | Forward and reverse nest primers for third fusion PCR for amplification of the *FgHSP90* repression construct |
| FgHSP90-3N pzear | TCTTGGAGAAGGCGCTGTAGAACTT |
| HYG-F1 | GGCTTGGCTGGAGCTAGTGGAGG | Forward and reverse primers for amplification of *P_zear_* with hygromycin resistance gene cassette from the *P_zear_-GzmetE* strain |
| zear-r2 | CATGGTTACTTTCGTTCTCTCTGGTC |
| pIGPAPA-sGFP | GTGAGCAAGGGCGAGGAGCTG | Forward primer for amplification of the GFP-HYG construct from pIGPAPA vector |
| FgHSP90-5F GFP | CCAGCGCTGGTGGTACTTTCTCC | Forward and reverse primers for amplification of 5′-flanking region for Gfp tagging *FgHSP90* with tail for the hygromycin resistance gene cassette fusion |
| FgHSP90-5R GFP | gaacagctcctcgcccttgctcacGTCGACCTCCTCCATGGC |
| FgHSP90-3F GFP | cctccactagctccagccaagccCCTTTTTCGGATGGGGTGTAACTC | Forward and reverse primers for amplification of 3′-flanking region for Gfp tagging *FgHSP90* with tail for the hygromycin resistance gene cassette fusion |
| FgHSP90-3R GFP | TGCACGCTAGTCCAAGAATACGG |
| FgHSP90-gfpF | GTACCTCCATCATCCTCCACCTCAA | Forward and reverse nest primers for third fusion PCR for amplification of Gfp tagging *FgHSP90* construct |
| FgHSP90-gfpR | CCTGATCCTGAGGGGTCTGACAG |
| HSP90-rt-F | AGTTCCGTGCCATCCTCTTCGT | For realtime-PCR of *HSP90* |
| HSP90-rt-R | GCGGACGTAGAGCTTGATGTTGTT |
| STUA-rt-F | CAGAACGGAAATGATGGTGGACTC | For realtime-PCR of *STUA* |
| STUA-rt-R | ATTGGAAAGAGGCTGGTGAAGGT |
| HTF1-rt-F | GGAAGAAGAGCTGAGGTGGGACAT | For realtime-PCR of *HTF1* |
| HTF1-rt-R | TGGAAGTTGGGGGAGCGGT |
| REN1-rt-F | ACGACAGACTTGAATCGCCTGACA | For realtime-PCR of *REN1* |
| REN1-rt-R | TATCGTGCCACATCGTATCCAGCA |
| ABAA-rt-F | ACTCAGGAAGCTTTGACCACGGC | For realtime-PCR of *ABAA* |
| ABAA-rt-R | GGGCTCTGGTAGGGGTTGACAGTA |
| WETA-rt-F | GTTCCAGGTACTCCCACTGCCAT | For realtime-PCR of *WETA* |
| WETA-rt-R | ACGTTCTCGTCGCGCTTTGGT |
| UBH-rt-F | GTTCTCGAGGCCAGCAAAAAGTCA | For realtime-PCR of *UBH* |
| UBH-rt-R | CGAATCGCCGTTAGGGGTGTCTG |
| 16768-rt-F | CACCGCTGGCTCTGACATTGA | For realtime-PCR of FGSG_16768 |
| 16768-rt-R | GCAGCGAGCTAGGACGAGTGAAT |
| 10816-rt-F | CCTTCCAACCCAGCTATGACACC | For realtime-PCR of FGSG_10816 |
| 10816-rt-R | CTGCACCATCCTGAGAGAAGCG |
| 08768-rt-F | TCGTGGCGGTATGCAAATCTTC | For realtime-PCR of FGSG_08768 |
| 08768-rt-R | AAAATTTGCATACCACCACGAAGG |
| 03247-rt-F | CACATACCCATCCGTCAGCAAACT | For realtime-PCR of FGSG_03247 |
| 03247-rt-R | GATGCGGAACCAGTCAAGAATGTC |
| 00766-rt-F | AGAAGGCCATGGATGCTGAAGTT | For realtime-PCR of FGSG_00766 |
| 00766-rt-R | GAAGATCTCGGAGGCGTTGGA |
| 02316-rt-F | CATCTTCGCCCCCGTTCTGA | For realtime-PCR of FGSG_02316 |
| 02316-rt-R | GTGCGAGCATCTGTTTTGAGGAA |

**Table S2. Primers used in this study.**


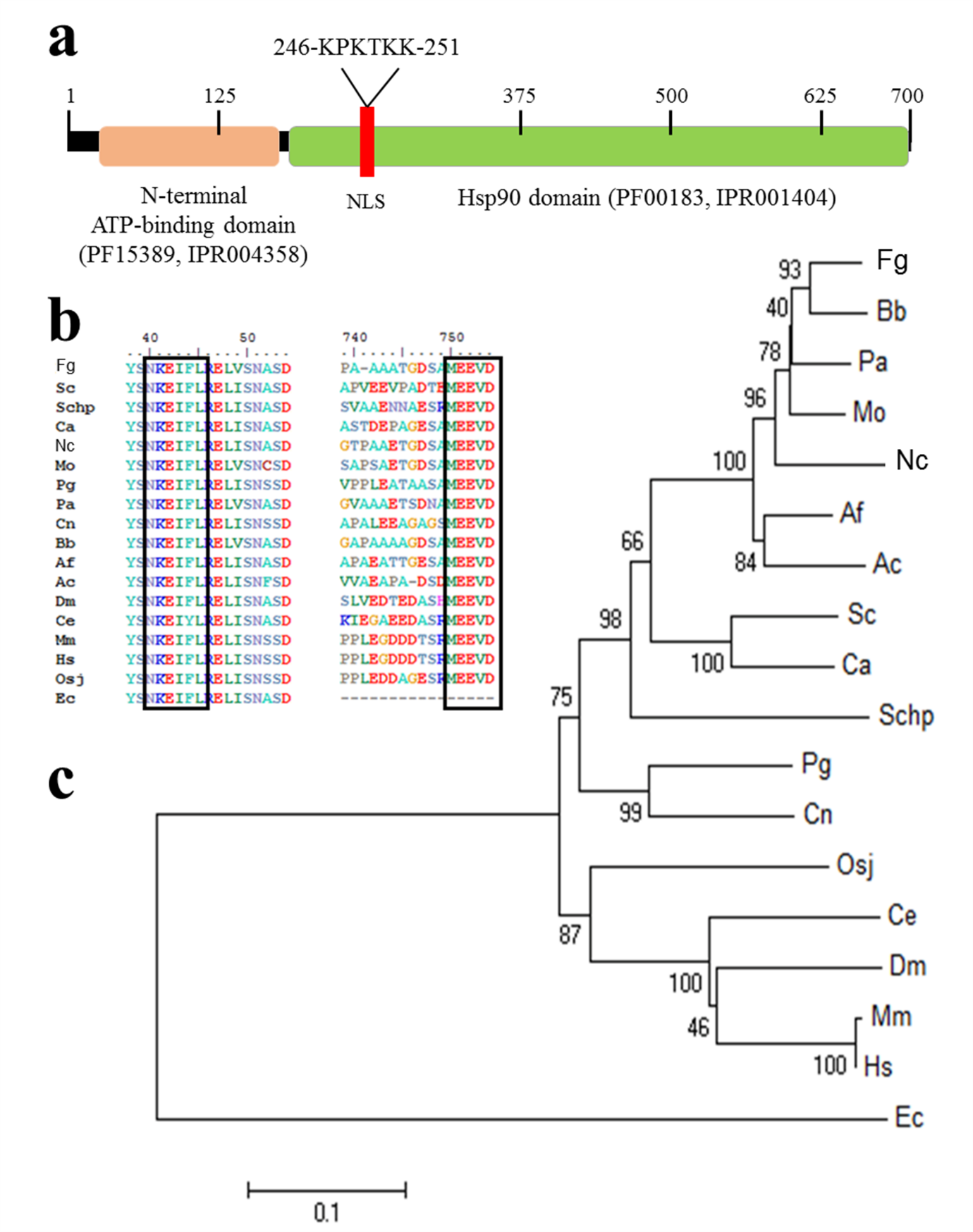


**Figure S1.** **Molecular characterisation of *Fg*Hsp90.** (**a**) Schematic presentation of the conserved protein domains of *Fg*Hsp90 in *F. graminearum* by InterPro database (http://www.ebi.ac.uk/interpro/) and the HMMPham database (<http://pfam.sanger.ac.uk/>). NLS, nuclear localisation signal predicted by NLStradamus[^9^](#_ENREF_9). (**b**) The eukaryotic evolutionary Hsp90 protein motifs, NKEIFL and MEEVD, are also conserved in *F. graminearum* based on the alignment of *Fg*Hsp90 amino acid sequence with other representative species, including bacteria, yeasts, filamentous fungi, plant, and animals. Fg, *Fusarium graminearum*; Bb, *Beauveria basiana*; Pa, *Podospora anserina*; Mo, *Magnaporthe oryzae*; Nc, *Neurospora crassa*; Af, *Aspergillus fumigatus*; Ac, *Ajellomyces capsulatus*; Sc, *Saccharomyces cerevisiae*; Ca, *Candida albicans*; Schp, *Schizosaccharomyces pombe*; Pg, *Puccinia graminis*; Cn, *Cryptococcus neoformans*; Osj, *Oryza sativa japonica*; Ce, *Caenorhabditis elegans*; Dm, *Drosophila melanogaster*; Mm, *Mus musculus*; Hs, *Homo sapiens*; Ec, *Escherichia coli*. (**c**) Phylogenetic tree of homologs of the Hsp90 from representative species constructed using amino acid sequences comparison.


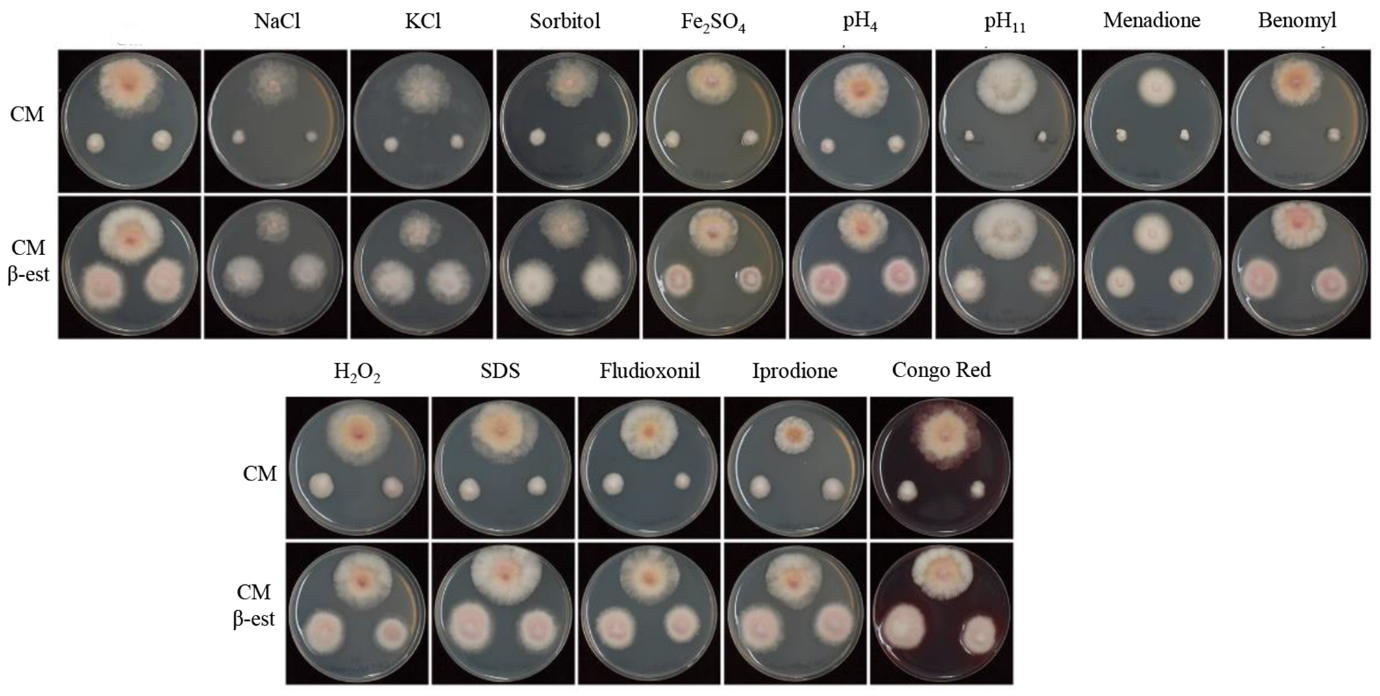


**Figure S2.** **The observation of various stresses response in the repression of *FgHSP90* mutants (HK226).** The pictures were taken at 3 days after inoculation.


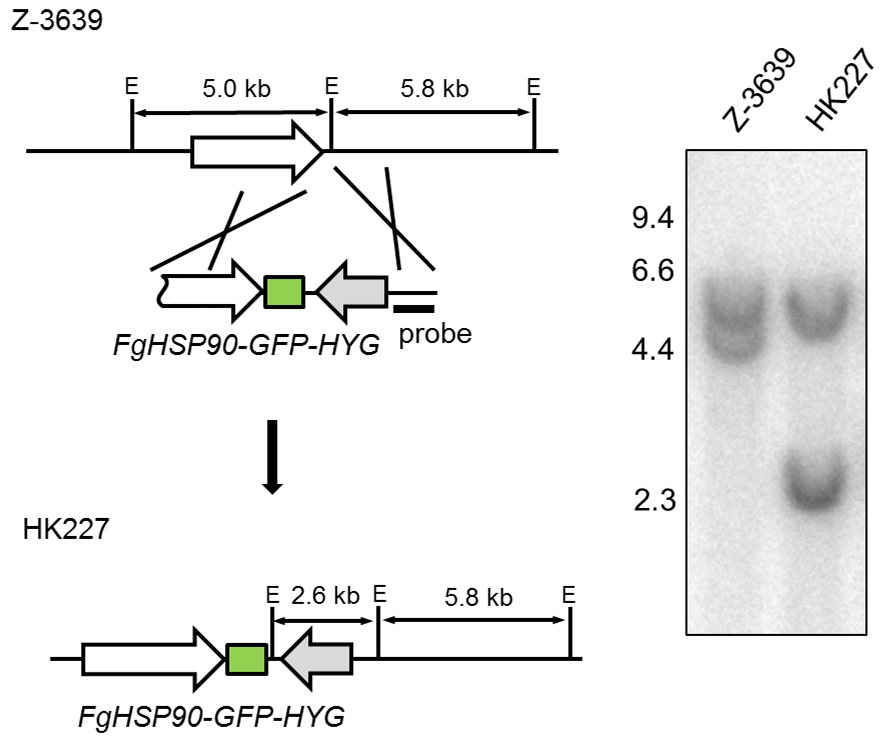


**Figure S3.** Schematic illustrating the strategy for fusion of Gfp to *Fg*Hsp90. Left panel: Strategy used to fuse *GFP* to the 3ʹ end of *FgHSP90*. Right panel: Southern blot analysis confirming genetic construct. Lane 1, wild-type Z-3639 strain; Lane 2, *FgHSP90*-*GFP* strain (HK227). Sizes of the DNA standards (kb) are indicated to the left of the blot. E, EcoRV.


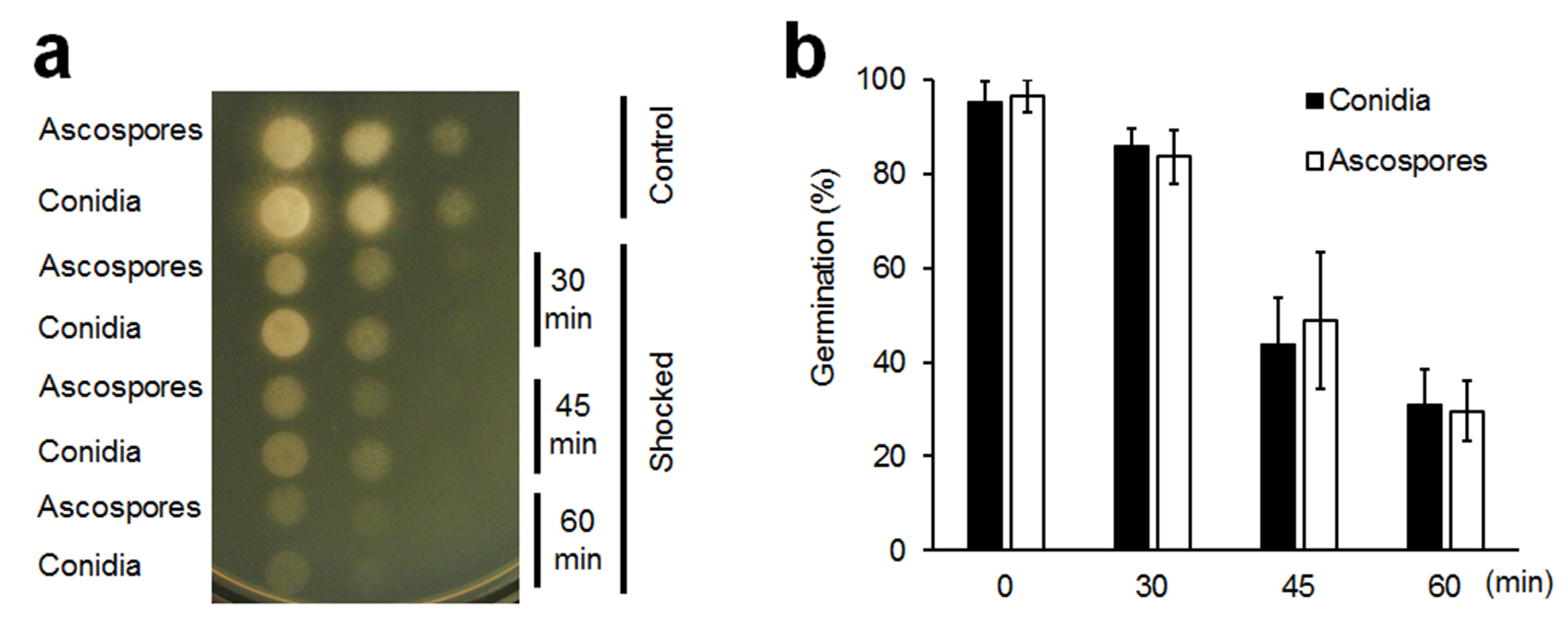


**Figure S4.** Heat shock response of the sexual and asexual spores in *F. graminearum*. (**a**) Serial dilutions of wild-type conidia and ascospores were point-inoculated onto CM medium. The heat shock treatment was conducted at 42 ^o^C for 30, 45, and 60 min, respectively. (**b**) Germination assay. Germination rate was measured from optimal and heat shock treatment assays 12 h after inoculation. One hundred conidia were assessed for each strain with three biological replicates.

**Supplementary References**

1. Bowden, R. L. & Leslie, J. F. Sexual recombination in *Gibberella zeae*. *Phytopathology* **89**, 182-188, (1999).

2. Hong, S.-Y. *et al.* Functional analyses of two syntaxin-like SNARE genes, *GzSYN1* and *GzSYN2*, in the ascomycete *Gibberella zeae*. *Fungal Genet. Biol.* **47**, 364-372, (2010).

3. Lee, J., Son, H., Lee, S., Park, A. R. & Lee, Y.-W. Development of a conditional gene expression system using a zearalenone-inducible promoter for the ascomycete fungus *Gibberella zeae*. *Appl. Environ. Microbiol.* **76**, 3089-3096, (2010).

4. Son, H., Lee, J., Park, A. R. & Lee, Y.-W. ATP citrate lyase is required for normal sexual and asexual development in *Gibberella zeae*. *Fungal Genet. Biol.* **48**, 408-417, (2011).

5. Son, H., Min, K., Lee, J., Raju, N. B. & Lee, Y.-W. Meiotic silencing in the homothallic fungus *Gibberella zeae*. *Fungal Biol.* **115**, 1290-1302, (2011).

6. Lee, J., Lee, T., Lee, Y. W., Yun, S. H. & Turgeon, B. G. Shifting fungal reproductive mode by manipulation of mating type genes: obligatory heterothallism of *Gibberella zeae*. *Mol. Microbiol.* **50**, 145-152, (2003).

7. Min, K. *et al.* Peroxisome function is required for virulence and survival of *Fusarium graminearum*. *Mol. Plant-Microbe Interact.* **25**, 1617-1627, (2012).

8. Son, H. *et al.* AbaA regulates conidiogenesis in the ascomycete fungus *Fusarium graminearum*. *PloS One* **8**, e72915, (2013).

9. Ba, A. N. N., Pogoutse, A., Provart, N. & Moses, A. M. NLStradamus: a simple Hidden Markov Model for nuclear localization signal prediction. *BMC Bioinformatics* **10**, 202, (2009).
